# Emergence of a multidrug-resistant *Pseudomonas fulva* clinical isolate co-harboring *tmexCD3*–*toprJ3*, *bla*_OXA-1_, and *bla*_IMP-45_ on a transferable megaplasmid

**DOI:** 10.3389/fcimb.2026.1722020

**Published:** 2026-02-16

**Authors:** Ziheng Wang, Jie Li, Yingying Li, Zihao Chen, Enze Ren, Peng Zhang

**Affiliations:** 1 Department of Laboratory Medicine, Yijishan Hospital of Wannan Medical College, Wuhu, Anhui, China; 2 Central Laboratory, The First Affiliated Hospital of Wannan Medical College, Wuhu, China; 3 School of Electronics and Information Engineering, Anhui University, Hefei, Anhui, China

**Keywords:** *bla*_IMP-45_, *bla*_OXA-1_, megaplasmid, multidrug resistance, *Pseudomonas fulva*, *tmexCD3–toprJ3*

## Abstract

**Introduction:**

*Pseudomonas fulva* is a non-fermentative bacterium with both environmental adaptability and pathogenic potential that has been increasingly detected in clinical infections in recent years. This study reports the first clinical *P. fulva* (PF1) isolate carrying *tmexCD3-toprJ3*, *bla*_OXA-1_, and *bla*_IMP-45_ resistance genes, which was recovered from the urine of a patient with a urinary tract infection.

**Methods:**

Species identification was performed using whole-genome sequencing and average nucleotide identity analysis, with phylogenetic placement determined by 16S rRNA. Genomic analysis identified resistance genes and plasmid structures, while plasmid transferability was assessed experimentally. Biofilm formation, stress tolerance, and virulence were evaluated using crystal violet staining, stress assays, and the *Galleria mellonella* model.

**Results:**

PF1 exhibited resistance to carbapenems, cephalosporins, quinolones, and an elevated tigecycline MIC of 256 mg/mL, while remaining susceptible to polymyxins. The strain harbors a 449-kb transferable megaplasmid (pPF1), containing resistance genes *bla*_IMP-45_, *bla*_OXA-1_, and a mutated *tmexCD3-toprJ3* efflux pump(T1827G and T1830C). Phylogenetic analysis showed >99% genomic similarity to clinical isolates from eastern China. PF1 demonstrated strong biofilm formation (OD620 = 3.73 ± 0.14), stress tolerance, and moderate virulence in *Galleria mellonella*.

**Discussion:**

This study reveals the potential of *P. fulva* to acquire multidrug resistance and adapt to clinical environments, underscoring the need for enhanced surveillance of resistance genes in atypical *Pseudomonas* species.

## 1 Introduction

*Pseudomonas fulva* is a member of the *Pseudomonas putida* complex and has long attracted attention due to its distinctive metabolic capabilities. From a One Health perspective, *P. fulva* has significant metabolic and biotechnological value in environmental remediation and industrial applications. Previous studies have shown that *P. fulva* can efficiently degrade various pesticides and environmental pollutants. For instance, the *P. fulva* strain P31, isolated from agricultural soil, is capable of degrading the pyrethroid insecticide *d*-phenothrin, offering potential for eco-friendly pesticide pollution control in sustainable agriculture (Yang et al., 2018). In addition, *P. fulva* often functions as a consortium member in the degradation of organophosphate pesticides, synergistically breaking down toxic compounds such as phorate, thereby contributing to the maintenance of environmental homeostasis in soil ecosystems (Jariyal et al., 2018). Environmental isolates of *P. fulva* can also utilize typical pollutants—including bisphenol A, estradiol, and nonylphenol—as carbon sources, demonstrating promising potential for applications in industrial wastewater treatment and the remediation of endocrine-disrupting contaminants (Mireisz et al., 2024). In terms of metabolic pathways, the *Pseudomonas* genus has been shown to degrade complex aromatic and alkaloid compounds such as nicotine and nornicotine, with studies revealing molecular modules and regulatory networks that may also be utilized by *P. fulva* (Huang et al., 2020). Therefore, this species represents not only an important microbial resource for environmental remediation and industrial biotechnology but also a potential environmental reservoir of antimicrobial resistance genes, increasing its relevance and risk in clinical infections.

Although *P. fulva* plays an important role in environmental biotechnology, it has long been regarded as a rare opportunistic pathogen in clinical settings. However, its infection spectrum is expanding from trauma- and device-associated infections to community-acquired infections, including urinary tract infections (UTIs). Since the first reported case of *P. fulva* meningitis in 2010 (Almuzara et al., 2010), the first community-acquired cystitis caused by *P. fulva* without prior trauma was documented in 2022 (Stark, 2022), suggesting that this species is not confined to nosocomial contexts. Subsequent cases, including septic shock in elderly hospitalized patients, indicate that *P. fulva* can cause severe infections in immunocompromised hosts (Kohno et al., 2023).

In terms of antimicrobial resistance, the *Pseudomonas* genus has been widely reported to harbor multiple β-lactamases, particularly metallo-β-lactamases (MBLs) such as *bla*_IMP-45_, which are predominantly disseminated via IncP-2 plasmids among clinical isolates in China (Zhang et al., 2021; Ma et al., 2025). In addition, *bla*_OXA-1_, a class D oxacillinase more commonly found in Enterobacterales, has also been identified in *Pseudomonas*, indicating a further expansion of its resistance spectrum (Wang et al., 2023). Tigecycline, a novel glycylcycline antibiotic, exhibits potent *in vitro* activity against various resistant pathogens such as methicillin-resistant *Staphylococcus aureus* (MRSA) and carbapenem-resistant Gram-negative bacteria and is widely used in the treatment of severe infections, including complicated skin and soft tissue infections, intra-abdominal infections, and community-acquired pneumonia (Moghnieh et al., 2020). In recent years, the emergence of tigecycline resistance has drawn increasing concern. Beyond intrinsic efflux pump systems, the discovery of the mobile *tmexCD–toprJ* gene cluster has established *Pseudomonas* species as important reservoirs and disseminators of tigecycline resistance determinants (Mai et al., 2023). Of particular concern is that transferable plasmids carrying both *bla*_VIM-24_ and *tmexCD3–toprJ3* have been identified in clinical *P. fulva* isolates (Liu et al., 2025), indicating that this species can integrate and disseminate resistance determinants against multiple “last-resort antibiotics.”.

Building on this background, we systematically characterized the urine-derived clinical isolate *P. fulva* PF1. We performed species identification and phylogenetic placement and combined antimicrobial susceptibility testing with whole-genome sequencing (WGS) to define its resistome and genetic contexts. We further conducted structural annotation of the resistance-bearing megaplasmid pPF1 and experimentally verified its transferability. Notably, pPF1 co-harbored *bla*_IMP-45_, *bla*_OXA-1_, and a *tmexCD3*–*toprJ3* gene cluster with the T1827G/T1830C variants, and it was stably transferred to a *Pseudomonas* recipient in conjugation assays. These findings suggest that atypical *Pseudomonas* species may integrate and disseminate a “carbapenem/tigecycline-associated” resistance module via mobile megaplasmids, thereby increasing the complexity of clinical treatment and surveillance.

## 2 Materials and methods

### 2.1 Clinical strain isolation and identification

**Figure 1:**
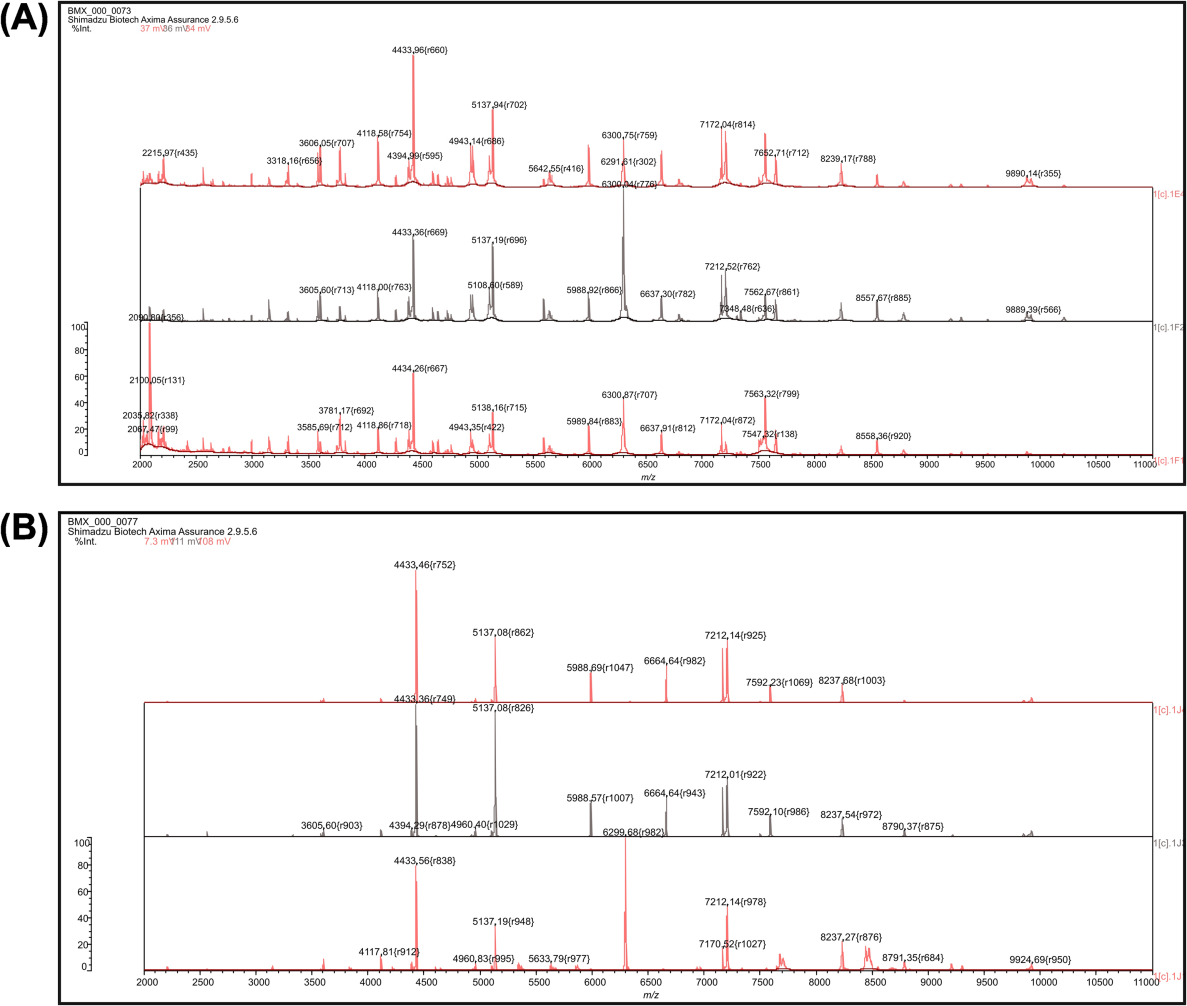
Matrix-assisted laser desorption/ionization time-of-flight mass spectrometry (MALDI-TOF MS) spectra. **(A)** MALDI-TOF MS analysis of PF1, performed in triplicate. **(B)** MALDI-TOF MS analysis of *Pseudomonas putida* ATCC 49128, performed in triplicate.

Fresh urine samples were collected from a patient with a clinically diagnosed UTI in Anhui Province who had comorbid renal calculi, hydropneumothorax, chronic obstructive pulmonary disease (COPD), and lacunar cerebral infarction and who had undergone closed thoracic drainage. The urine samples were inoculated onto blood agar plates and incubated at 37°C for 18 h (**Supplementary Figure S1**). The isolate was preliminarily identified as *P. putida* by matrix-assisted laser desorption/ionization time-of-flight mass spectrometry (MALDI-TOF MS) (bioMérieux, Marcy-l’Étoile, France) (**Figures 1A, B**), and species confirmation was achieved by WGS followed by average nucleotide identity (ANI) analysis using the online tool JSpeciesWS (Richter et al., 2016).

### 2.2 Antimicrobial susceptibility testing

The antimicrobial susceptibility of the isolate was evaluated using the Vitek 2 Compact system (bioMérieux, Marcy-l’Étoile, France). Minimum inhibitory concentrations (MICs) of tigecycline and eravacycline were determined with the broth microdilution method, while susceptibility to ceftazidime–avibactam was assessed using the Kirby–Bauer disk diffusion method. *Escherichia coli* ATCC 25922 and *Pseudomonas aeruginosa* ATCC 27853 were used as the quality control strains. The MIC values for *Pseudomonas* spp. were interpreted according to the breakpoint recommendations of the Clinical and Laboratory Standards Institute (CLSI M100, 2024) and the European Committee on Antimicrobial Susceptibility Testing (EUCAST, 2024).

### 2.3 Whole-genome sequencing and annotation

Genomic DNA from the isolate was extracted using the NucleoBond^®^ HMW DNA Kit (MN, Düren, Germany). The DNA concentration and purity were measured using Qubit 4.0 (Thermo Fisher, Waltham, MA, USA) and NanoDrop, while integrity was verified with agarose gel electrophoresis. Sequencing libraries were prepared and subjected to paired-end sequencing (PE150) on the MGI DNBSEQ-T7 platform. To obtain complete assemblies, long-read sequencing was additionally performed using Oxford Nanopore Technologies (ONT, Oxford, UK). Short reads were quality-filtered using fastp (v0.23.0), and ONT long reads were filtered using Fastplong (v0.2.2). Hybrid *de novo* assembly was conducted with Unicycler (v0.5.1), followed by polishing with NextPolish (v1.4.1) using short-read data (Wick et al., 2017; Hu et al., 2020). Plasmid circularization was determined automatically by Unicycler (v0.5.1) based on the overlap between the contig termini. The circularized plasmid sequences were further confirmed by alignment against closely related plasmids available in the NCBI database. Functional annotation was performed using the Kyoto Encyclopedia of Genes and Genomes (KEGG), the Virulence Factor Database (VFDB), and the Comprehensive Antibiotic Resistance Database (CARD) (Kanehisa et al., 2017; Liu et al., 2022; Alcock et al., 2023).

### 2.4 Phylogenetic analysis

Whole-genome sequences of PF1 were compared with the reference *Pseudomonas* strains available in the NCBI database. A 16S rRNA phylogenetic tree was constructed using MEGA software, and visualization was refined using the online tool ChiPlot (Xie et al., 2023).

### 2.5 Plasmid analysis

Plasmid and mobile genetic element contexts were analyzed using MOB-suite, PLSDB, ISfinder, and ICEberg (Siguier et al., 2006; Robertson and Nash, 2018; Galata et al., 2019). Antimicrobial resistance genes were identified using CARD and ResFinder (Florensa et al., 2022). Plasmid maps were generated with the online tool Proksee, and resistance gene synteny was visualized using Easyfig (Sullivan et al., 2011; Grant et al., 2023).

### 2.6 Stress resistance analysis

To evaluate bacterial survival under host-mimicking stress conditions (i.e., bile salts, osmotic stress, and acidic stress), logarithmic-phase cultures were diluted 1:100 in Luria–Bertani (LB) broth containing different concentrations of bile salts, NaCl, or HCl and incubated at 37°C with shaking at 180 rpm. The OD_600_ was measured hourly using a UV spectrophotometer, and growth curves were plotted using GraphPad Prism 10.

### 2.7 *Galleria mellonella* infection model

Bacterial suspensions in the logarithmic phase were adjusted to 1 × 10^6^ and 1 × 10^7^ CFU/mL. *Galleria mellonella* larvae were divided into six groups (10 larvae per group) and injected with 10 μl of bacterial suspension or phosphate-buffered saline (PBS) into the last left proleg. The hypervirulent *Klebsiella pneumoniae* strain NTUH-K2044 served as the positive control, and PBS as the negative control. After infection, the larvae were incubated at 37°C in the dark, survival was monitored every 12 h, and Kaplan–Meier survival curves were plotted (Mai et al., 2023). All experiments were performed in triplicate.

### 2.8 Biofilm formation assay

Diluted cultures of PF1 in the logarithmic phase (OD_600_ = 0.6) were inoculated into 96-well polystyrene microtiter plates. After incubation, the medium was discarded, and the wells were washed three times with PBS, followed by staining with crystal violet. The wells were then rinsed with distilled water, and the bound dye was solubilized with 95% ethanol. The OD_620_ value was determined using a microplate reader. Each group included four replicates, and experiments were performed in triplicate.

### 2.9 Conjugation assay

PF1 was used as the donor strain, while *E. coli* J53 and a clinically isolated *P. aeruginosa* strain, PA1, were used as recipient strains to evaluate plasmid transferability. PA1 was resistant to polymyxin but remained susceptible to carbapenem antibiotics. Briefly, overnight cultures of the donor and recipient strains were mixed at a 1:1 ratio and spread onto LB agar plates, followed by incubation at 37°C overnight. Transconjugants were selected on Mueller–Hinton (MH) agar plates supplemented with either sodium azide (200 µg/mL) and imipenem (4 µg/mL) or with polymyxin (4 µg/mL) and imipenem (4 µg/mL). Transconjugants were identified using MALDI-TOF MS (bioMérieux, Marcy-l’Étoile, France). The presence of the *bla*_IMP-45_ resistance gene in the transconjugants was confirmed by PCR (*bla*_IMP-45_F: AACACGGCTTGGTGGTTCTTGTAA; *bla*_IMP-45_R: GAGCGACGAATCTCCTATGTCACTATG). Antimicrobial susceptibility testing was performed using the Vitek 2 Compact system. The conjugation frequency was calculated by dividing the number of transconjugants by the number of recipient cells.

### 2.10 Efflux pump inhibition assay

Carbonyl cyanide *m*-chlorophenylhydrazone (CCCP) was employed to evaluate the contribution of efflux pumps to tigecycline resistance. MH broth media containing tigecycline or eravacycline at serial concentrations ranging from 1 to 512 µg/mL were prepared using the twofold serial dilution method. The final concentration of CCCP was maintained at 10 µg/mL in both antibiotic-supplemented and antibiotic-free MH broth media. The experiment was divided into three groups: the antibiotic–CCCP combination group, the antibiotic-only group, and the CCCP-only group. Each group was assayed in triplicate.

## 3 Results

### 3.1 Identification and phylogenetic positioning of the clinical isolate

High-quality WGS was performed to support species identification and phylogenetic analyses. The complete chromosome (4,972,618 bp) showed 100% coverage with an average sequencing depth of 140.31× (**Supplementary Figure S2**).

**Figure 2:**
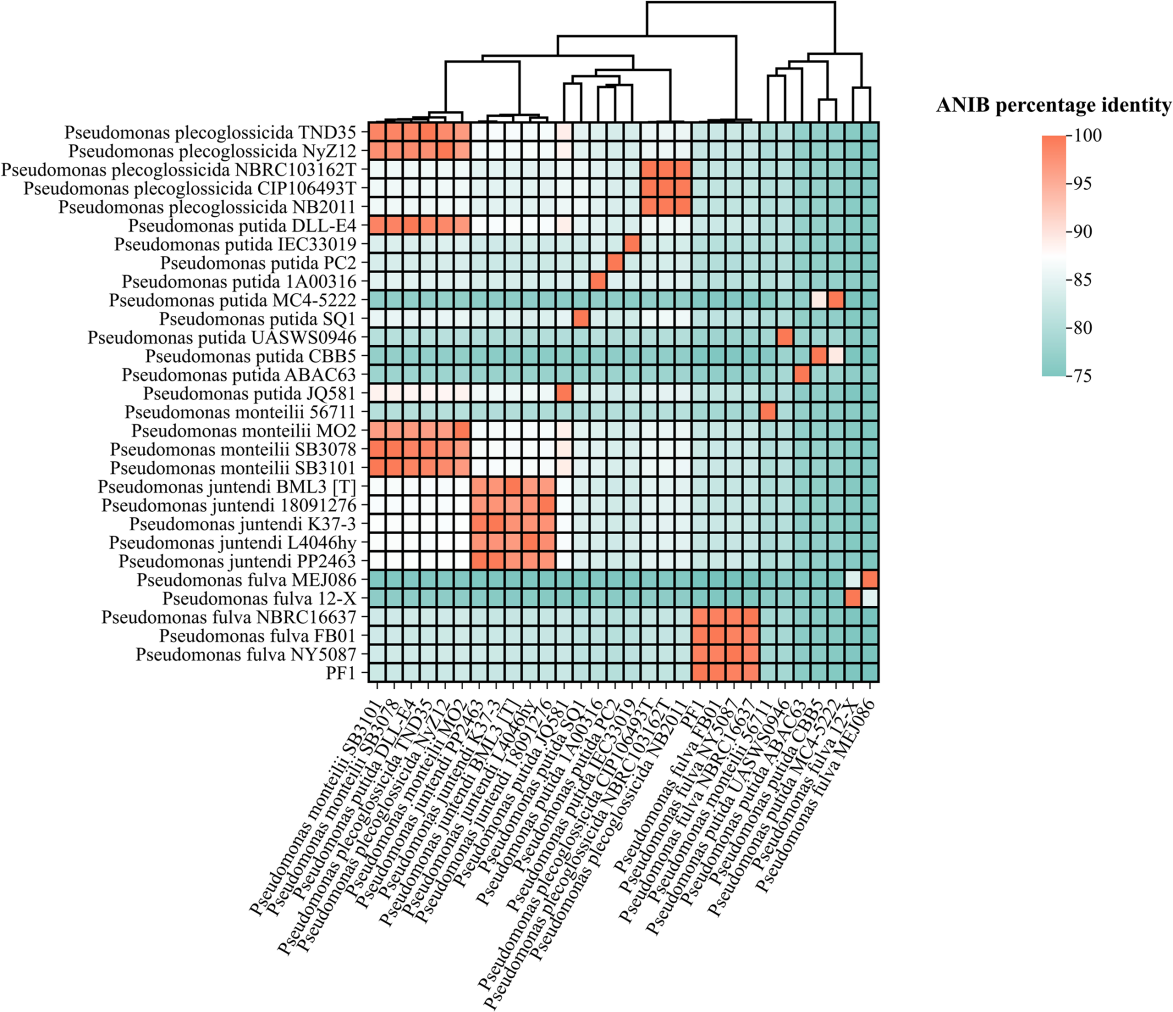
Heatmap of the ANIb (average nucleotide identity using BLAST) scores between PF1 and 29 reference genomes. The *color gradient* represents ANIb similarity (from 90% in *light yellow* to 100% in *dark red*).

The ANIb (average nucleotide identity using BLAST) heatmap (**Figure 2**) showed that the target strain PF1 shared 99.81% genomic similarity to the clinical isolate *P. fulva* FB01 (GCA_049315045.1), which was obtained from a patient in Jinhua, Zhejiang Province, China, in 2024. Moreover, PF1 exhibited ANI values of 99.17% and 99.2% with *P. fulva* NY5087 (GCA_039634495.1), isolated from the urine of a patient with prostate cancer in Henan, China, in 2019, and *P. fulva* NBRC16637 (GCA_015679205.1), recovered from the bile of a liver transplant patient in the same province in 2019, respectively. These results indicate that PF1 shares extremely high genomic similarity with clinically isolated *P. fulva* strains, well above the species delineation threshold (95%–96%). In contrast, PF1 exhibited markedly lower ANIb values (<83%) with closely related species such as *P. putida*, *P. monteilii*, and *P. juntendi*, demonstrating clear species boundaries. Collectively, these findings confirm that PF1 is taxonomically classified as *P. fulva*.

**Figure 3:**
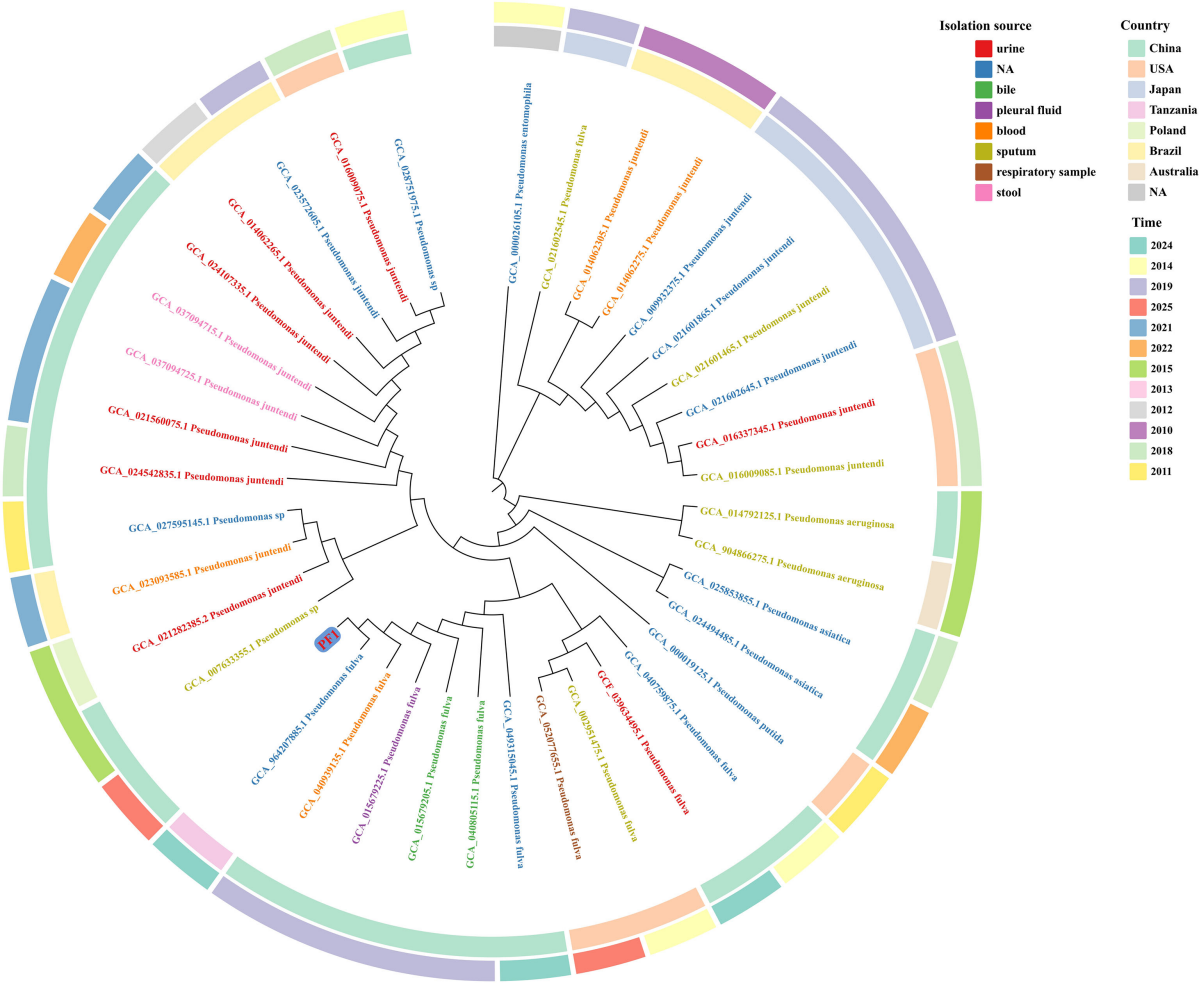
Phylogenetic tree of clinical *Pseudomonas fulva* isolates based on 16S rRNA sequences. In the phylogenetic tree, the *leaf colors* denote different isolation sources, whereas the *inner* and *outer concentric rings* indicate the country and year of isolation, respectively.

To further verify the phylogenetic position of PF1, we incorporated 12 clinical *P. fulva* genomes and related *Pseudomonas* species sequences available in the NCBI database to construct a 16S rRNA-based phylogenetic tree (**Figure 3**). The results showed that PF1 clustered tightly with FB01, forming a highly supported branch, and was closely grouped with other clinical *P. fulva* isolates, including NY5087 and NBRC16637. In contrast, PF1 was clearly separated from closely related species such as *P. juntendi*, *P. putida*, and *P. asiatica*.

### 3.2 Antimicrobial susceptibility profile and distribution of resistance genes

Antimicrobial susceptibility testing (**Table 1**) revealed that PF1 exhibited extensive resistance to multiple clinically relevant antibiotics. The strain was resistant to carbapenems, third- and fourth-generation cephalosporins, aminoglycosides, fluoroquinolones, and sulfonamides but remained susceptible only to colistin. The tigecycline MIC was elevated at 256 μg/mL. Notably, the MIC for tigecycline was markedly elevated, indicating a clinically significant level of resistance.

**Table 1:** Minimum inhibitory concentrations (MICs) of PF1 and the associated resistance genes.

Antibiotic class	Antimicrobial resistance gene(s)	Antimicrobial agent	MIC^a^ (μg/mL)
β-Lactams	*bla*_OXA-1_ *bla*_IMP-45_	Ticarcillin–clavulanate	**>128 (R)**
		Piperacillin–tazobactam	**>128 (R)**
		Cefoperazone–sulbactam	**>64 (R)**
		Ceftazidime–avibactam^b^	**R**
		Ticarcillin	**>128 (R)**
		Piperacillin	**>128 (R)**
		Aztreonam	16 (I)
		Ceftazidime	**>64 (R)**
		Cefepime	**>32 (R)**
Carbapenems	*bla*_OXA-1,_ *bla*_IMP-45_	Imipenem	**>16 (R)**
		Meropenem	**>16 (R)**
Aminoglycosides	*aph(3')-Ia* *aac(6')-Ib3*(T304C) *armA*	Amikacin	**>64 (R)**
		Tobramycin	**>16 (R)**
Quinolones	*qnrVC6*	Levofloxacin	**>8 (R**)
		Ciprofloxacin	**>4 (R)**
		Norfloxacin	**>16 (R)**
Tetracyclines	*tmexC3* *tmexD3*(T1827G, T1830C) *TOprJ3*	Tetracycline^c^	8 (NA)
		Doxycycline^c^	8 (NA)
		Minocycline^c^	8 (NA)
Glycylcyclines	*tmexC3* *tmexD3*(T1827G, T1830C) *TOprJ3*	Tigecycline^c^	256 (NA)
		Eravacycline^c^	8 (NA)
Sulfonamides	*sul1*	Trimethoprim–sulfamethoxazole^c^	>320 (NA)
Polymyxins		Colistin	≤0.5 (S)

*R*, resistant; *I*, Intermediate; *S*, susceptible; *NA*, not available.

a Interpretations were based on CLSI M100 (2024) and EUCAST (2024), when available.

b Ceftazidime–avibactam was tested using the Kirby–Bauer disk diffusion method.

c Tetracycline, doxycycline, minocycline, tigecycline, eravacycline, and trimethoprim–sulfamethoxazole have no established clinical breakpoints for *Pseudomonas* spp. in either the Clinical and Laboratory Standards Institute (CLSI) or EUCAST guidelines; therefore, only the MIC values were reported and no susceptibility categorization (S/I/R) was applied.

The bold values indicate that the minimum inhibitory concentration (MIC) is higher than the clinical breakpoint set by CLSI M100 (2024) or EUCAST (2024), and the corresponding strain is resistant to the antimicrobial agent.

**Figure 4:**
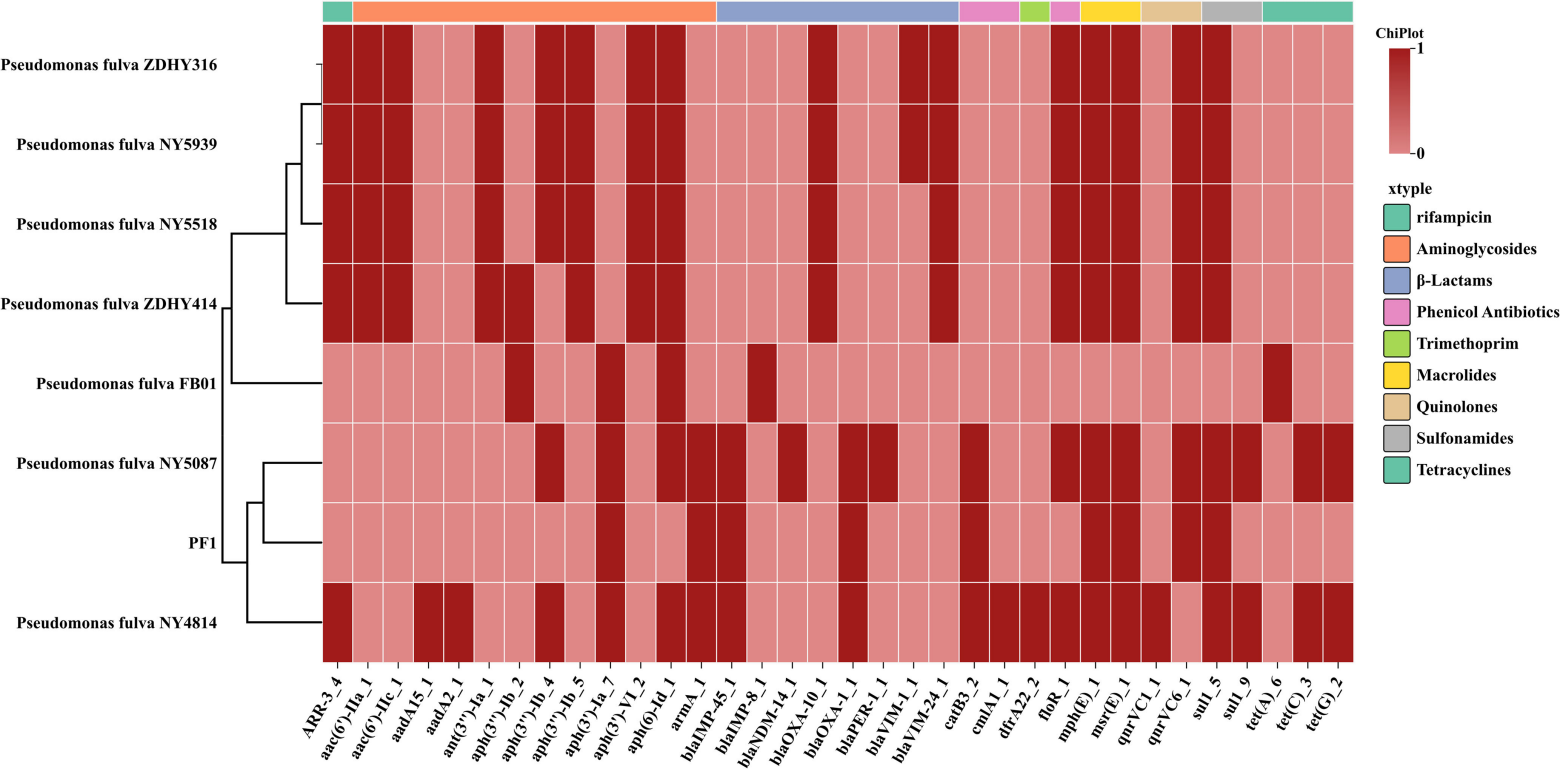
Heat map comparing the drug resistance genes carried by PF1 and all clinical isolates of *Pseudomonas fulva* in the NCBI database.

Resistance gene analysis (**Figure 4**) showed that PF1 carries the β-lactamase genes *bla*_IMP-45_ and *bla*_OXA-1_, conferring resistance to carbapenems and certain broad-spectrum cephalosporins. The *armA* gene mediates high-level resistance to aminoglycosides. In addition, PF1 harbors *catB3*, *floR*, *msr(E)*, *qnrVC1*, and *sul1*, which collectively correspond to its broad-spectrum resistance phenotype observed in the antimicrobial susceptibility testing.

Importantly, PF1 harbors the *tmexCD3*–*toprJ3* efflux pump gene cluster with two point mutations (T1827G and T1830C), the functional relevance of which remains to be clarified. To provide functional support for an efflux-mediated phenotype, we performed an efflux pump inhibition assay using CCCP coupled with MIC determination. In the presence of CCCP, the MIC of tigecycline decreased from 256 to 64 μg/mL, and that of eravacycline decreased from 8 to 2 μg/mL, representing a fourfold reduction for both agents. These data indicate that active efflux contributes to the elevated MICs of tigecycline and eravacycline in PF1, consistent with the presence of the *tmexCD3*–*toprJ3* cluster; however, the specific contribution of the T1827G/T1830C substitutions requires further confirmation using mutation-resolved approaches.

### 3.3 Structural characteristics and genetic context of the resistance gene-carrying plasmid

**Figure 5:**
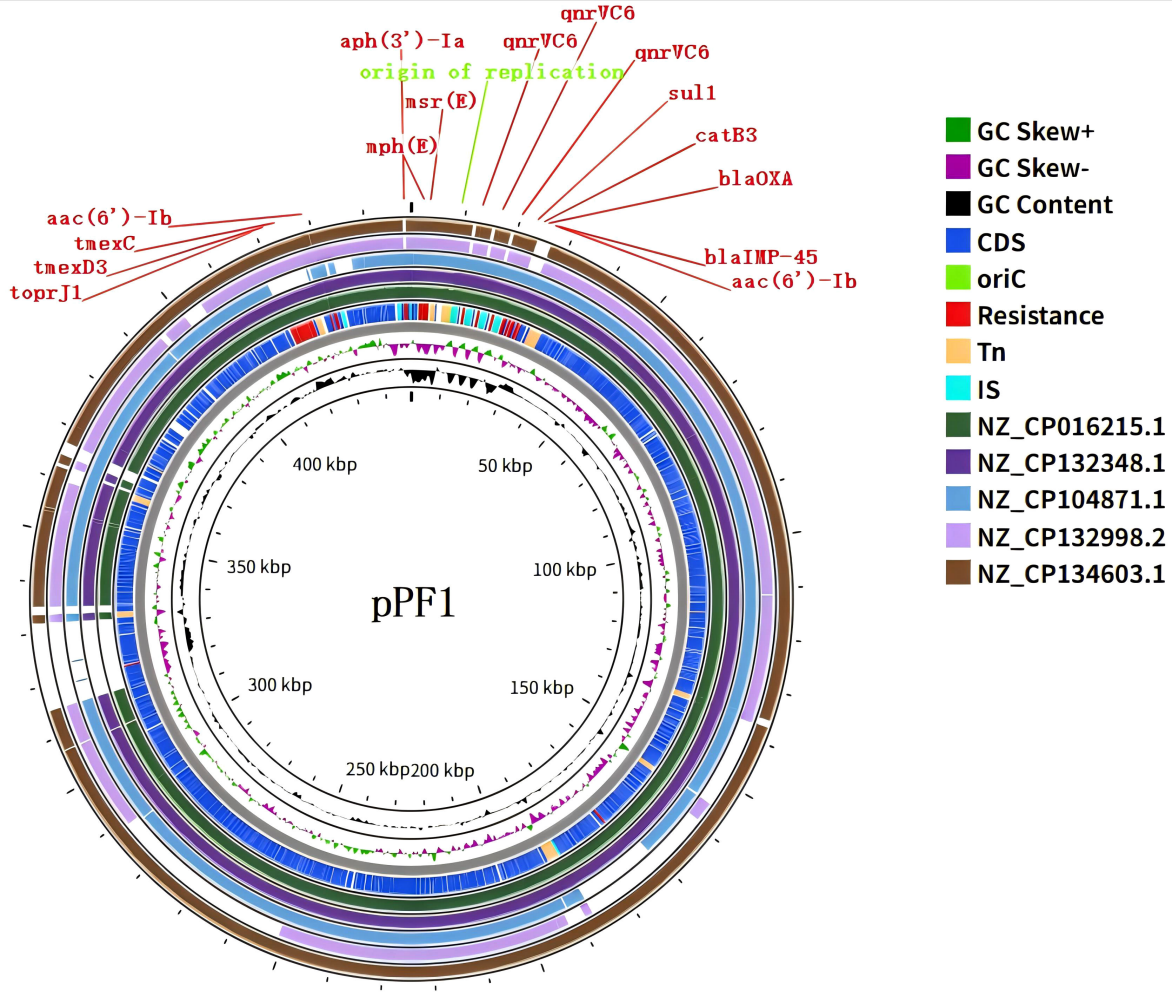
Map of the 448,885-bp megaplasmid carrying the *tmexCD3–toprJ3*, *bla*_OXA-1_, and *bla*_IMP-45_ genes. From *innermost to outermost*, the *circles* represent the GC content, the GC skew, and the genes carried by plasmid pPF1 compared with plasmids from *P. aeruginosa* (CP016215.1), *P. fulva* (CP132348.1), *P. aeruginosa* (CP104871.1), *P. aeruginosa* (CP132998.2), and *P. putida* (CP134603.1). The drug resistance genes carried by plasmid pPF1 are highlighted in *red*.

A large plasmid, designated pPF1, was identified in PF1, with a total length of 448,885 bp and an average GC content of 56.02%. The sequencing data showed complete coverage of the plasmid with an average depth of approximately 168.85× (**Supplementary Figure S3**). The multidrug resistance region of pPF1 harbors a diverse set of resistance determinants, including *aac(6')-Ib*, *sul1*, *bla*_IMP-45_, *bla*_OXA-1_, three tandemly repeated copies of *qnrVC6*, *aph(3')-Ia*, and the *tmexCD3*–*toprJ3* efflux pump gene cluster (**Figure 5**). Analysis of the plasmid backbone revealed the presence of replication and stability modules, including the rpeM replication initiation protein and the parAB partitioning system, as well as conjugative transfer-related gene clusters encoding a type IV secretion system and tra genes (**Supplementary Table S1**). Overall, the structural features of pPF1 are consistent with the pJBCL41-like megaplasmid backbone reported previously. However, because it cannot be assigned to any known incompatibility group, pPF1 was classified as an untypable megaplasmid.

Conjugation assays showed that the plasmid carried by PF1 could not be transferred to *E. coli* J53. In contrast, stable transconjugants were obtained when a clinical isolate of *P. aeruginosa* PA1 was used as the recipient strain, with a frequency of 5.89 × 10^−7^ transconjugants per donor cell. The resulting transconjugant was designated as TPA1. This finding indicates that the plasmid is more readily disseminated among *Pseudomonas* species through horizontal transfer. Antimicrobial susceptibility testing further confirmed that acquisition of the plasmid led to marked alterations in the resistance phenotype of the recipient strain. Specifically, the parental PA1 strain was susceptible to carbapenems, whereas the transconjugant TPA1 exhibited high-level resistance to both imipenem and meropenem, with MIC values exceeding 16 μg/mL. In addition, TPA1 shifted from susceptibility to resistance to multiple β-lactam/β-lactamase inhibitor combinations and cephalosporins (**Supplementary Table S2**), a change that is likely attributable to the plasmid-borne *bla*_IMP-45_ resistance gene. To further verify the molecular basis of this phenotypic shift, PCR amplification followed by agarose gel electrophoresis was performed, which revealed a specific band of the expected size corresponding to *bla*_IMP-45_ in the transconjugant strain. These results confirm that the resistance gene was horizontally transferred together with the plasmid and stably expressed in the recipient strain PA1.

**Figure 6:**
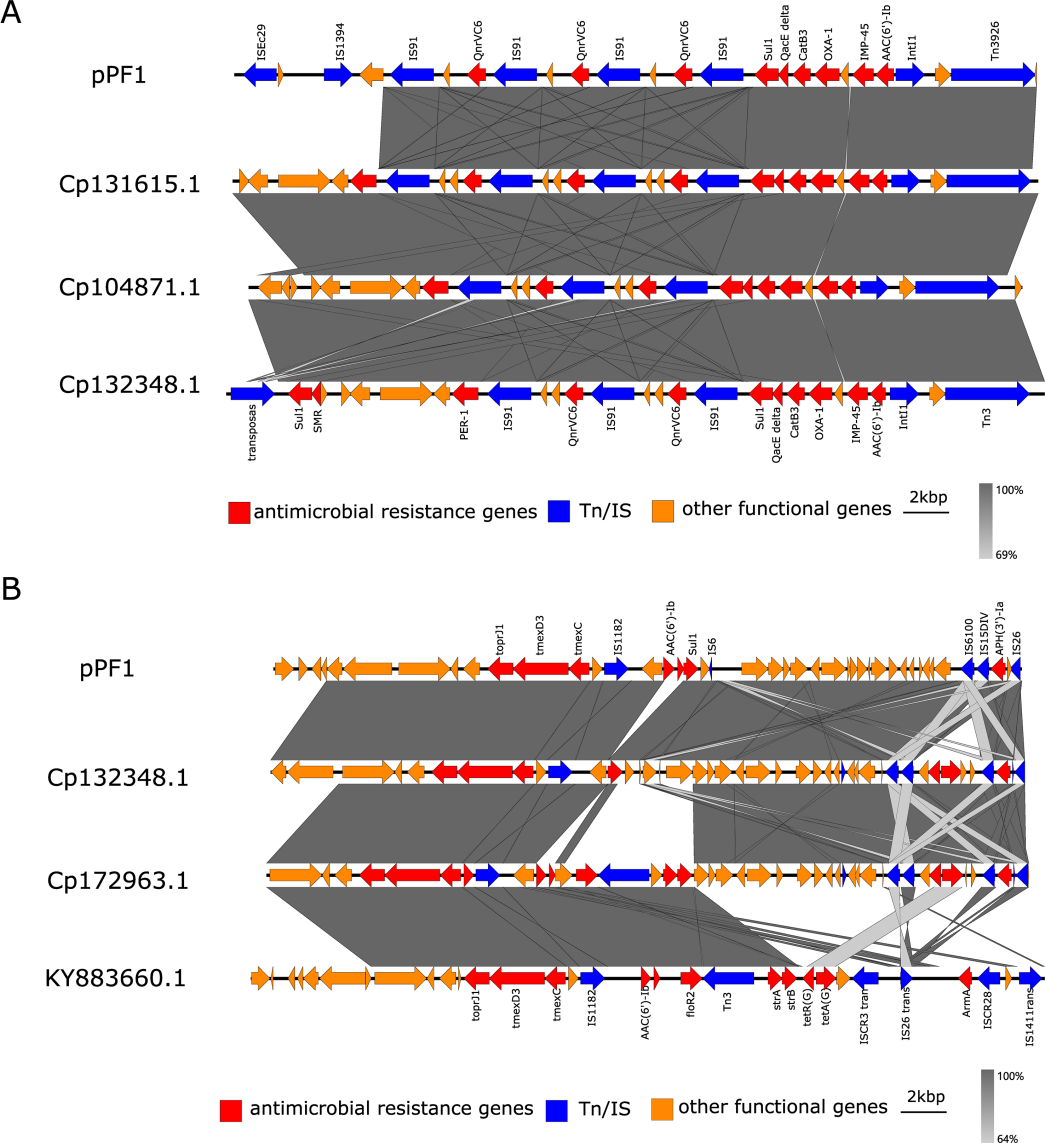
Linear comparison of the drug resistance genes. **(A)** Linear comparison of *bla*_IMP-45_ and *bla*_OXA-1_ on plasmid pPF1. **(B)** Linear comparison of *tmexCD3*–*toprJ3* on plasmid pPF1.

Linear comparative analysis revealed that the resistance island of this plasmid is composed of multiple functional modules, exhibiting a distinct mosaic structural pattern. In the upstream region, a tandemly arranged segment containing alternating *qnrVC6* genes and *IS91* transposases was observed, which is characteristic of the *IS91* family-mediated rolling-circle transposition (**Figure 6A**). Immediately downstream, a class 1 integron core region was identified, containing *aac(6')-Ib* and *bla*_IMP-45_, accompanied by the typical *qacEΔ1–sul1* 3' conserved segment. The intI1 integrase and its flanking sequences formed a canonical class 1 integron structure, within which *aac(6')-Ib* and *bla*_IMP-45_ were located and connected to the *qacEΔ1*–*sul1* terminal framework. Adjacent to this region, *bla*_OXA-1_ and *catB3* were also identified. This integron not only accounts for the resistance of PF1 to aminoglycosides and carbapenems but also demonstrates its ability to capture and accumulate new gene cassettes. Notably, transposable elements such as *Tn3926* transposase, resolvase, *ISEc29*, and *IS1394* were identified surrounding the integron, suggesting a strong potential for interplasmid or interspecies mobilization. In addition, the downstream region of the plasmid harbors the *tmexCD3*–*toprJ3* efflux pump module, flanked by *ISCfr1*, *IS6100*, and a secondary *qacEΔ1–sul1* unit, forming an efflux pump + integron terminus + IS composite structure (**Figure 6B**). Furthermore, a *TetR/AcrR* family transcriptional regulator protein was annotated adjacent to the *tmexCD3*–*toprJ3* module, which indicates that the expression of this efflux system may be subject to regulatory control (**Supplementary Table S3**).

### 3.4 Biological phenotypes and virulence characteristics

**Figure 7:**
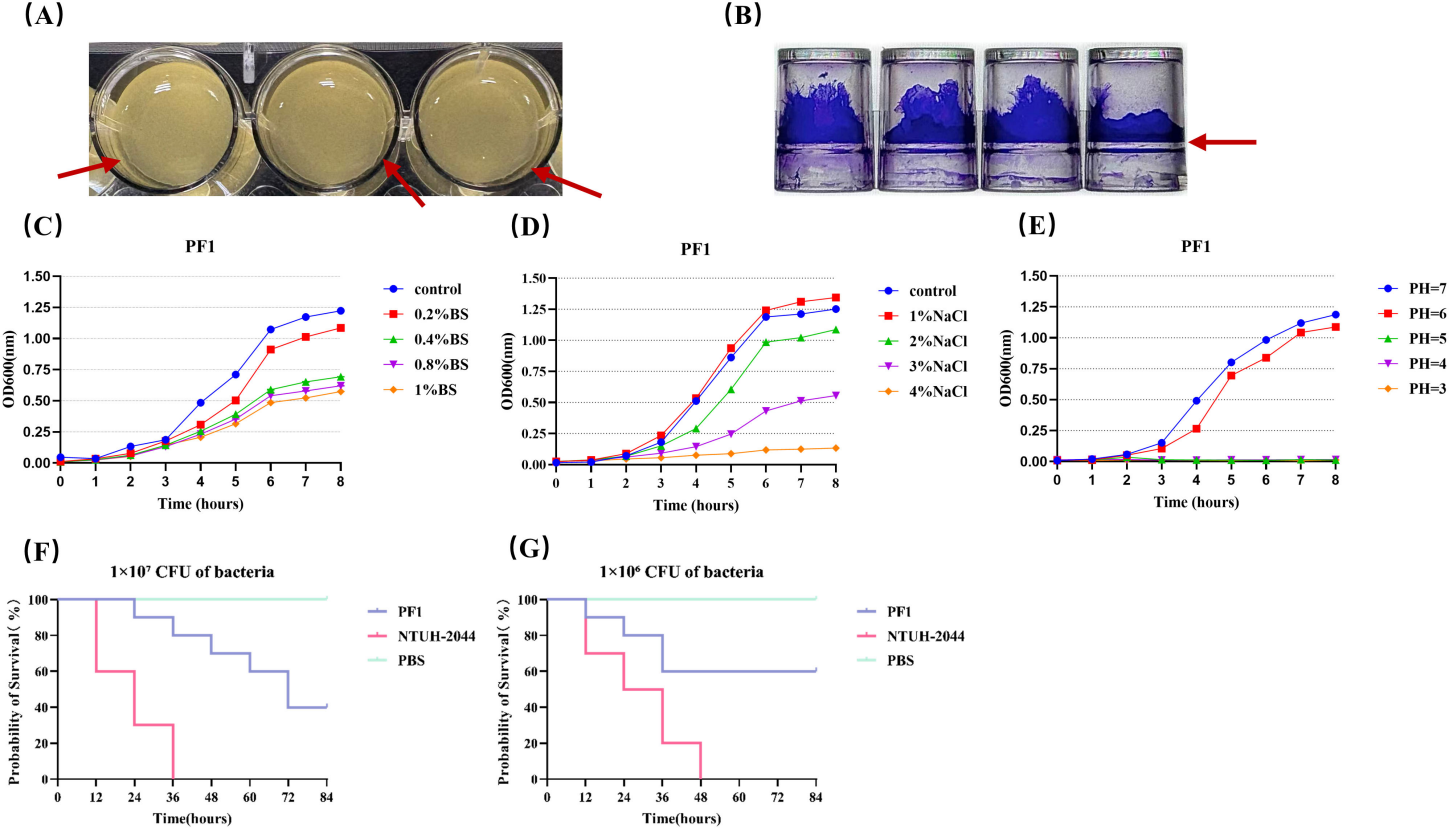
**(A)** Top view of the pellicle formed by the *P. fulva* strain PF1 after 24 h of static incubation. **(B)** Biofilm formation of *P. fulva* PF1 after 24 h was observed with the crystal violet assay. **(C–E)** Growth curves of *P. fulva* PF1 in different bile salt environments, different NaCl environments, and different acidic environments. **(F, G)** Survival curve of **(G)***mellonella* bacterial infection. The positive control group: hvKP NTUH-K2044; negative control group: phosphate-buffered saline (PBS).

Regarding the biological phenotypes, PF1 formed a distinct pellicle structure at the air–liquid interface under static culture conditions (**Figures 7A, B**). The biofilm biomass was quantified using crystal violet staining, revealing an OD_620_ value of 3.73 ± 0.14 for PF1, which is indicative of strong surface adherence and colonization capacity. Consistent with this phenotype, Clusters of Orthologous Groups (COGs) and Gene Ontology (GO) annotations revealed an enrichment of genes associated with exopolysaccharide biosynthesis, flagellar assembly, and adhesion. These genomic features align with the observed biofilm-forming ability of PF1 and likely contribute to host colonization and urinary tract adherence.

In terms of environmental stress tolerance, PF1 maintained robust growth under bile salt and NaCl stress conditions (**Figures 7C–E**). Functional genomic annotation revealed the presence of multiple stress response- and efflux-related genes, including RND (resistance–nodulation–division) and MFS (major facilitator superfamily) family efflux pumps, Na^+^/H^+^ antiporters, and acid–base homeostasis regulators, which likely provide adaptive advantages in both urinary and environmental niches. Moreover, BacMet analysis identified several heavy metal resistance genes, such as *czcABC*, *copA*, *merA*, and *terC*/D, which confer tolerance to copper, zinc, and mercury ions. These findings suggest that PF1 may possess co-selection potential for antimicrobial resistance under complex environmental conditions.

Regarding virulence, VFDB annotation revealed that PF1 carries multiple virulence-associated factors, including T6SS components (hcp, tssB, and tssC), flagellar assembly genes (*flg* and *fli*), quorum-sensing regulators (lasR and rhlR), and iron acquisition systems (pvdL and pvdI). In the *G. mellonella* infection model, PF1 exhibited a moderate virulence phenotype, with lethality slightly lower than that of the hypervirulent control strain *K. pneumoniae* NTUH-K2044 (**Figures 7F, G**). This may be attributed to the fact that the virulence of PF1 is primarily driven by its biofilm-forming ability and competitive fitness for nutrients rather than by the production of potent toxins.

## 4 Discussion

*P. fulva* was originally identified in environmental niches such as soil, plant rhizospheres, and aquatic habitats, exhibiting remarkable metabolic plasticity and environmental adaptability. It participates in the biotransformation of complex pollutants, including aromatic hydrocarbons, phenolic compounds, and endocrine-disrupting chemicals, and has been repeatedly reported in contexts of industrial pollution control and environmental remediation (Jariyal et al., 2018; Yang et al., 2018). Conversely, sporadic clinical reports have shown that *P. fulva* can also cause human infections. However, its epidemiological features and antimicrobial resistance burden remain poorly characterized, and clinical understanding of this species is still in the early stages (Almuzara et al., 2010; Stark, 2022; Kohno et al., 2023).

Although PF1 was initially identified as *P. putida* by MALDI-TOF MS, whole genome-based analyses ultimately confirmed that it belongs to *P. fulva*. This discrepancy highlights an important limitation of routine MALDI-TOF identification for atypical or rarely encountered *Pseudomonas* species, as incomplete reference spectra and the high proteomic similarity within the *P. putida* group may lead to misclassification. In clinical practice, isolates reported as *P. putida* but exhibiting unusual antimicrobial resistance phenotypes should be further validated using genome-based approaches, which can improve diagnostic accuracy and support targeted surveillance.

PF1 exhibited resistance to multiple classes of antimicrobial agents, showing particularly high resistance to tigecycline, fluoroquinolones, and carbapenems. Previous studies have demonstrated that the *tmexCD*–*toprJ* gene family represents a recently emerged plasmid-mediated efflux pump system capable of actively exporting tetracyclines and tigecycline. This system has become one of the key mechanisms underlying tigecycline resistance in clinical bacterial isolates (Sun et al., 2023; Gao et al., 2025). Precisely because *tmexCD*–*toprJ* can disseminate across hosts via mobile genetic elements, it has been regarded in recent years as a priority target for surveillance of antimicrobial resistance spread (Dong et al., 2022b; Peng et al., 2024). In non-fermenting Gram-negative bacteria, this module is not merely an additional resistance determinant; rather, it may be integrated into an already intricate efflux network, thereby producing a more pronounced stepwise upward shift in MICs. In parallel, large-scale genomic and metagenomic analyses have suggested a close evolutionary link between *P. aeruginosa* and the origin of *tmexCD*–*toprJ*, which further strengthens an ecology-informed framework connecting environmental reservoirs to clinical settings (Shi et al., 2025). Accordingly, when we detected *tmexCD3*–*toprJ3* in PF1 and observed a fourfold MIC reduction upon efflux inhibition, its clinical relevance and dissemination risk are better interpreted within a combined framework of “intrinsic *Pseudomonas* efflux systems plus the influx of a mobile efflux module” rather than as an isolated gene detection event (Dong et al., 2022a).

In this study, PF1 exhibited a markedly elevated MIC for tigecycline, whereas the MIC for eravacycline was comparatively low. This phenotypic dissociation between two tetracycline derivatives is not an isolated observation. A large-scale clinical study of *Acinetobacter baumannii* isolates reported that, among tigecycline-resistant isolates, the MIC values for tigecycline were up to fourfold higher than those for eravacycline (Chen et al., 2024). Eravacycline is a member of the fluorocycline class and differs from tigecycline due to key structural modifications to the tetracycline core D ring, including the substitution of the dimethylamino group with a fluorine atom at the C7 position and the introduction of a pyrrolidinoacetamide side chain at the C9 position (Kunz Coyne et al., 2024). Previous *in vitro* studies have demonstrated that the activity of eravacycline is generally less affected by the classical tetracycline-specific efflux pumps Tet(A)/Tet(B) and the ribosomal protection proteins Tet(M)/Tet(O) (Falagas et al., 2025), suggesting that eravacycline may retain higher effective intracellular exposure in the presence of tetracycline resistance mechanisms and therefore exhibit lower MIC values than tigecycline.

On the plasmid pPF1, the regions surrounding *bla*_IMP-45_ and *bla*_OXA-1_ were flanked by multiple copies of *IS91* family transposases, *IS4* family element *ISEc29*, *IS30* family element *IS1394*, *Tn3926* transposase, and a class 1 integron consisting of intI1 and attI. These elements were accompanied by additional resistance genes, including *sul1*, *qacEΔ1*, *qnrVC6*, *catB3*, and *aac(6')-Ib*. Together, these mobile elements and gene cassettes form a characteristic “integron–*IS91* tandem–transposon composite segment,” which provides the genetic framework for the capture, assembly, and potential horizontal transfer of *bla*_IMP-45_ and *bla*_OXA-1_. Notably, on plasmid pPF1, the *tmexCD3–toprJ3* cluster is positioned adjacent to an *IS1182* family transposase and a LacI-type regulatory protein, forming an “efflux pump–IS composite unit” that facilitates modular mobilization. In this study, the coexistence of *bla*_IMP-45_, *bla*_OXA-1_, and *tmexCD3–toprJ3* on a single plasmid in a clinical *P. fulva* isolate suggests that resistance to both carbapenems and tigecycline, two last-line therapeutic agents, may be simultaneously compromised—substantially complicating clinical treatment strategies (Evans and Amyes, 2014; Zhang et al., 2021). In addition, multiple *qnrVC6*–*IS91* tandem modules were identified on pPF1, interspersed with multiple copies of *IS91* transposases exhibiting features of *IS91*-mediated rolling-circle replication. This mechanism enables replicative overrun and co-mobilization of adjacent DNA fragments, forming pseudo-composite transposition units that facilitate the rapid amplification of copy number and intra-/interhost transfer of quinolone resistance loci (Cross et al., 2025).

At present, systematic surveillance data on *P. fulva* in China remain extremely limited, with the majority of the reported cases originating from urine, bile, and blood samples in eastern and Central China. The close phylogenetic relationship between PF1 and the clinical isolates FB01, NY5087, and NBRC16637 revealed in this study suggests the possible existence of occult transmission routes in hospital or environmental settings. Considering the environmental nature of *P. fulva* and its adaptive potential in complex ecological niches, we interpreted these findings within a One Health framework. Functional annotation based on COG, GO, and KEGG analyses revealed that the PF1 genome is enriched in genes associated with signal regulation, defense and stress responses, membrane transport, and environmental adaptation (**Supplementary Figures S4–S6**). *In vitro* phenotypic assays further confirmed that PF1 maintained robust survival under stress conditions, including exposure to bile salts and osmotic pressure. Notably, we demonstrated that the resistance-bearing megaplasmid could establish stable transconjugants in *Pseudomonas* recipients, highlighting its ability for dissemination within *Pseudomonas* populations. Together, the convergence of genomic features, phenotypic adaptability, and plasmid transmissibility provides a plausible evidence base supporting a transmission continuum linking environmental reservoirs, the hospital ecological niche, and subsequent patient infections. Enhanced whole-genome monitoring of atypical *Pseudomonas* species—particularly those carrying *tmexCD–toprJ* and IMP- and OXA-type resistance determinants—is essential to prevent their establishment as reservoirs of multidrug resistance genes. Furthermore, for isolates identified as *P. putida* by MALDI-TOF MS, genomic ANI analysis and resistance gene profiling are recommended to avoid species misidentification.

## 5 Conclusion

This study reports the first clinical *P. fulva* isolate co-harboring *bla*_OXA-1_, *bla*_IMP-45_, and a mutated *tmexCD3*–*toprJ3* efflux pump on a transferable megaplasmid. The resistance genes are organized within a complex *integron*–*IS–Tn* composite structure. Phylogenetic analysis revealed a close relatedness between PF1 and clinical isolates from eastern China, indicating possible regional dissemination. The combination of multidrug resistance, robust biofilm formation, and stress tolerance likely facilitates persistence in hospital settings, underscoring the need for enhanced surveillance of atypical *Pseudomonas* species as emerging reservoirs of resistance.

## Data Availability

The datasets presented in this study can be found in online repositories. The names of the repository/repositories and accession number(s) can be found below: https://www.ncbi.nlm.nih.gov/, GCA_052907385.1 https://www.ncbi.nlm.nih.gov/, SRR36593758 https://www.ncbi.nlm.nih.gov/, SRR36593757.
