# Supplementary figures and images for: Emergence of a multidrug-resistant Pseudomonas fulva clinical isolate co-harboring tmexCD3–toprJ3, blaOXA-1, and blaIMP-45 on a transferable megaplasmid
Source: Front Cell Infect Microbiol. 2026 Feb 16;16:1722020. doi: 10.3389/fcimb.2026.1722020 (PMC12950786; doi:10.3389/fcimb.2026.1722020)

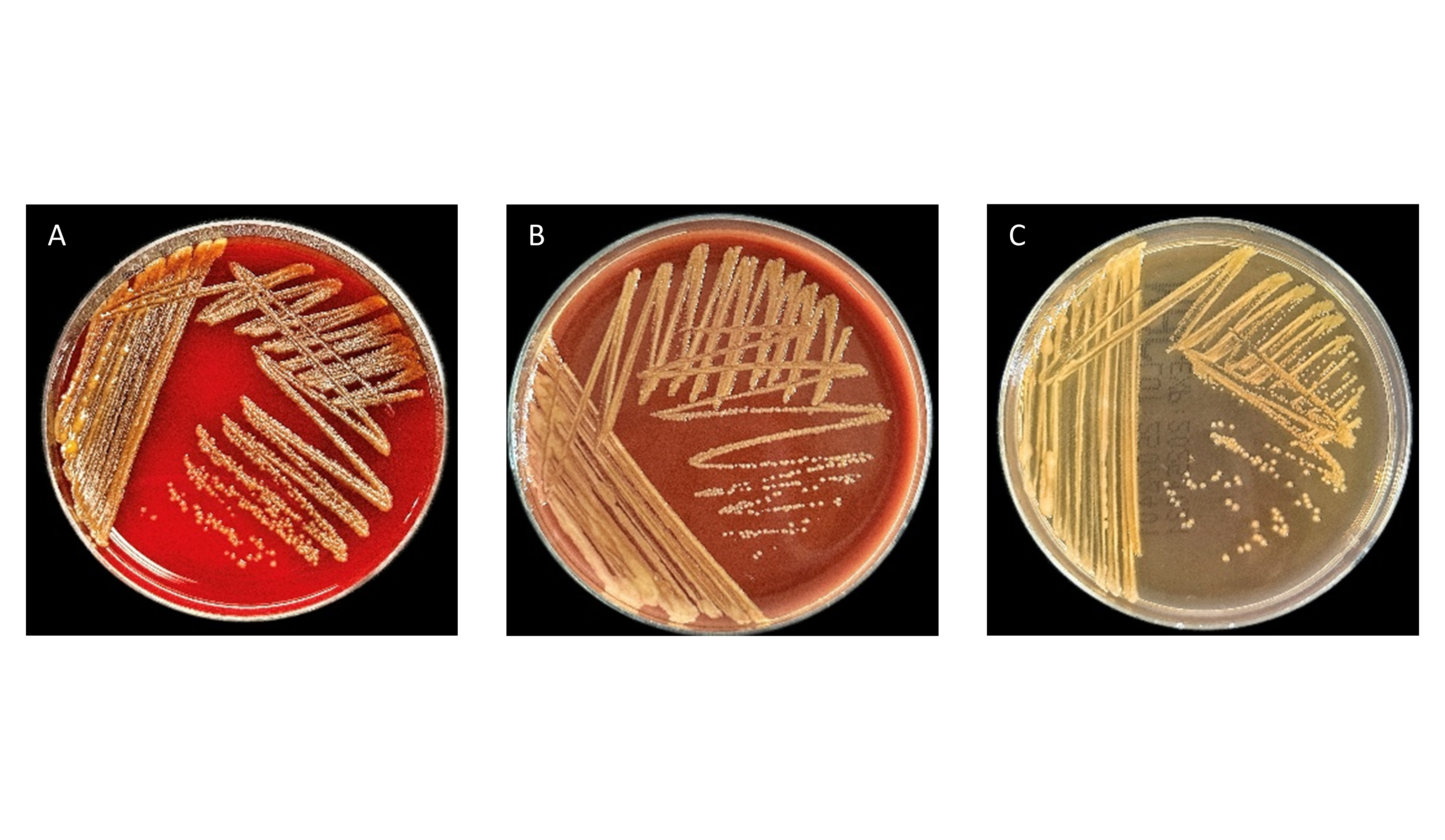

Supplement: Supplementary file 1 [file Image1.tif]

Gene Function Classification (GO)

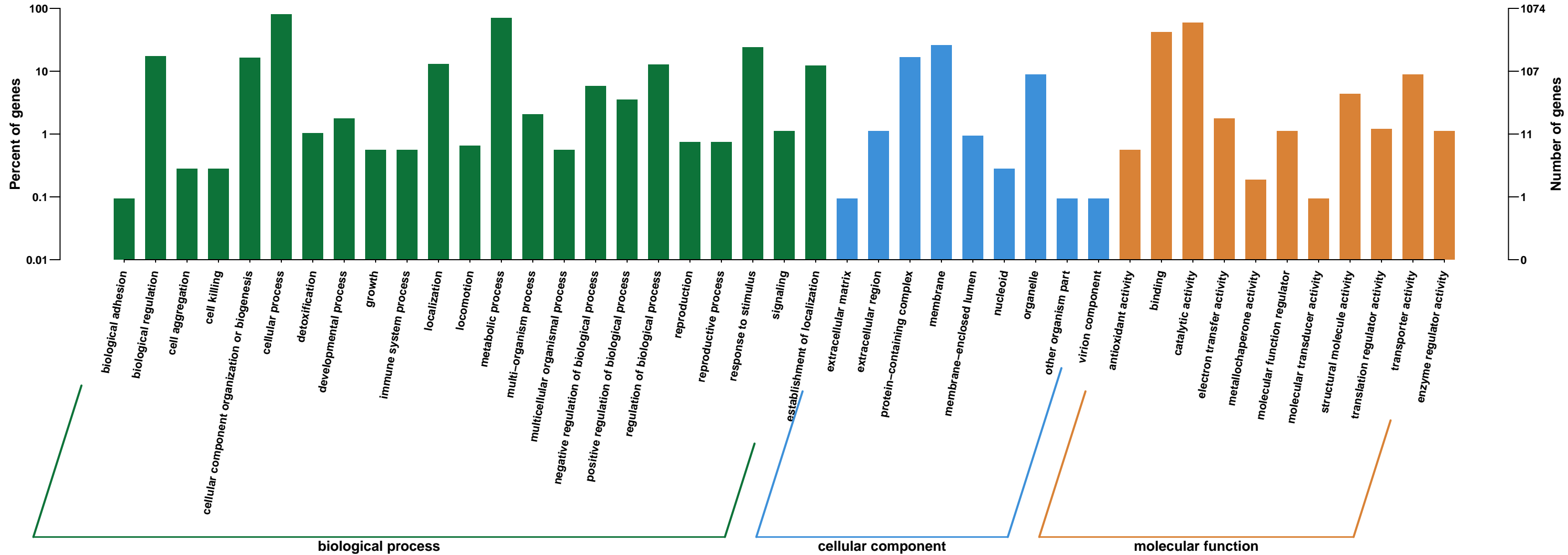

Supplement: Supplementary file 5 [file Image5.pdf]

# KEGG Pathway Classification

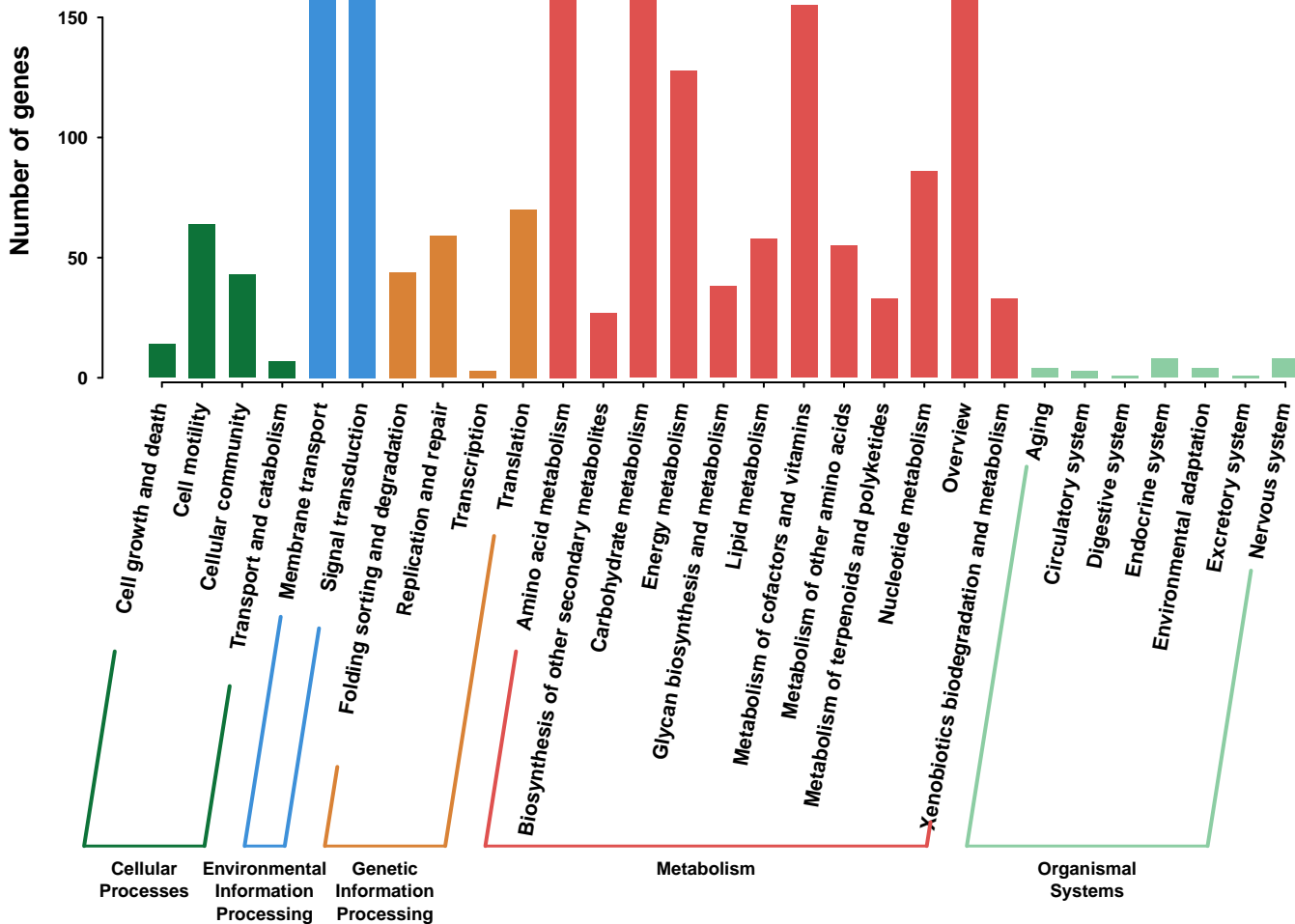

Supplement: Supplementary file 6 [file Image6.pdf]
