# Supplementary material for: Emergence of a multidrug-resistant Pseudomonas fulva clinical isolate co-harboring tmexCD3–toprJ3, blaOXA-1, and blaIMP-45 on a transferable megaplasmid
Source: Front Cell Infect Microbiol. 2026 Feb 16;16:1722020. doi: 10.3389/fcimb.2026.1722020 (PMC12950786; doi:10.3389/fcimb.2026.1722020)
Supplement: Supplementary file 2 [file Image2.pdf]

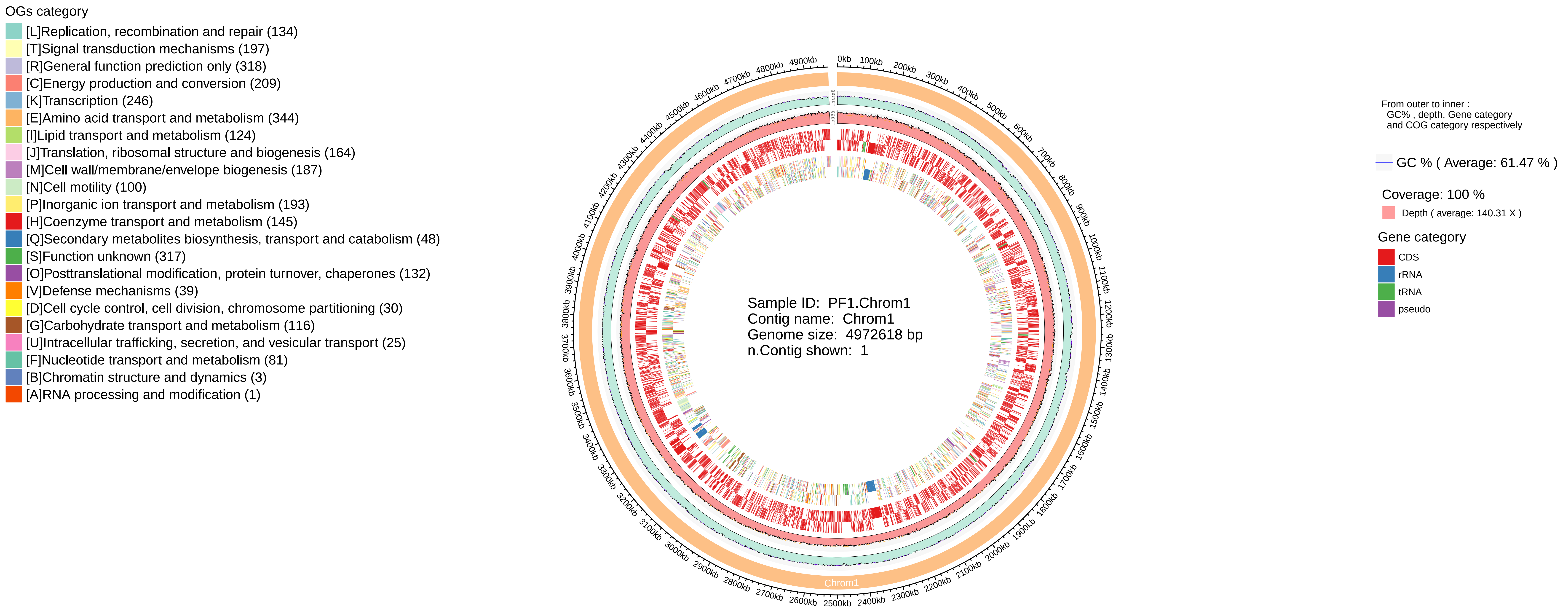
OGs category
[L]Replication, recombination and repair (134)
[T]Signal transduction mechanisms (197)
[R]General function prediction only (318)
[C]Energy production and conversion (209)
[K]Transcription (246)
[E]Amino acid transport and metabolism (344)
[I]Lipid transport and metabolism (124)
[J]Translation, ribosomal structure and biogenesis (164)
[M]Cell wall/membrane/envelope biogenesis (187)
[N]Cell motility (100)
[P]Inorganic ion transport and metabolism (193)
[H]Coenzyme transport and metabolism (145)
[Q]Secondary metabolites biosynthesis, transport and catabolism (48)
[S]Function unknown (317)
[O]Posttranslational modification, protein turnover, chaperones (132)
[V]Defense mechanisms (39)
[D]Cell cycle control, cell division, chromosome partitioning (30)
[G]Carbohydrate transport and metabolism (116)
[U]Intracellular trafficking, secretion, and vesicular transport (25)
[F]Nucleotide transport and metabolism (81)
[B]Chromatin structure and dynamics (3)
[A]RNA processing and modification (1)
Sample ID: PF1.Chrom1
Contig name: Chrom1
Genome size: 4972618 bp
n.Contig shown: 1
Chrom1
From outer to inner :
GC% , depth, Gene category
and COG category respectively
GC % ( Average: 61.47 % )
Coverage: 100 %
Depth ( average: 140.31 X )
Gene category
CDS
rRNA
tRNA
pseudo
