# Supplementary material for: Emergence of a multidrug-resistant Pseudomonas fulva clinical isolate co-harboring tmexCD3–toprJ3, blaOXA-1, and blaIMP-45 on a transferable megaplasmid
Source: Front Cell Infect Microbiol. 2026 Feb 16;16:1722020. doi: 10.3389/fcimb.2026.1722020 (PMC12950786; doi:10.3389/fcimb.2026.1722020)
Supplement: Supplementary file 3 [file Image3.pdf]

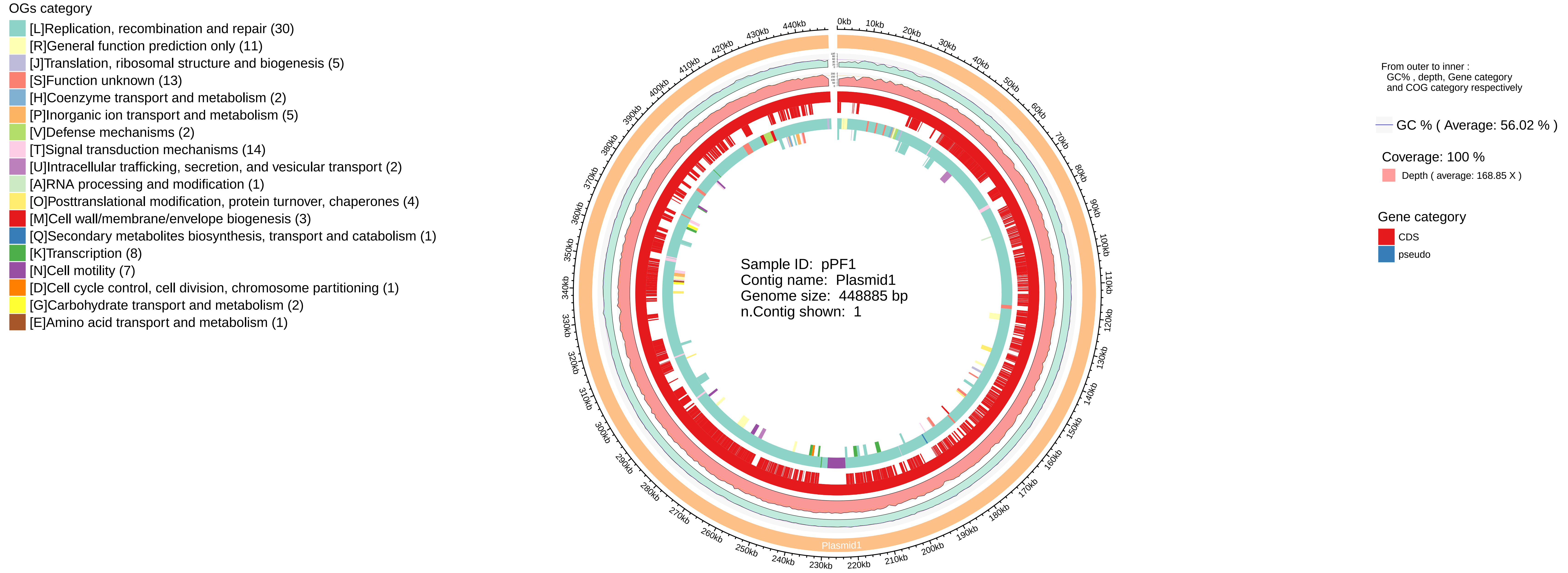
OGs category
[L]Replication, recombination and repair (30)
[R]General function prediction only (11)
[J]Translation, ribosomal structure and biogenesis (5)
[S]Function unknown (13)
[H]Coenzyme transport and metabolism (2)
[P]Inorganic ion transport and metabolism (5)
[V]Defense mechanisms (2)
[T]Signal transduction mechanisms (14)
[U]Intracellular trafficking, secretion, and vesicular transport (2)
[A]RNA processing and modification (1)
[O]Posttranslational modification, protein turnover, chaperones (4)
[M]Cell wall/membrane/envelope biogenesis (3)
[Q]Secondary metabolites biosynthesis, transport and catabolism (1)
[K]Transcription (8)
[N]Cell motility (7)
[D]Cell cycle control, cell division, chromosome partitioning (1)
[G]Carbohydrate transport and metabolism (2)
[E]Amino acid transport and metabolism (1)
Sample ID: pPF1
Contig name: Plasmid1
Genome size: 448885 bp
n.Contig shown: 1
Plasmid1
From outer to inner :
GC% , depth, Gene category
and COG category respectively
GC % (Average: 56.02 %)
Coverage: 100 %
Depth (average: 168.85 X)
Gene category
CDS
pseudo
