# Supplementary material for: Emergence of a multidrug-resistant Pseudomonas fulva clinical isolate co-harboring tmexCD3–toprJ3, blaOXA-1, and blaIMP-45 on a transferable megaplasmid
Source: Front Cell Infect Microbiol. 2026 Feb 16;16:1722020. doi: 10.3389/fcimb.2026.1722020 (PMC12950786; doi:10.3389/fcimb.2026.1722020)
Supplement: Supplementary file 4 [file Image4.pdf]

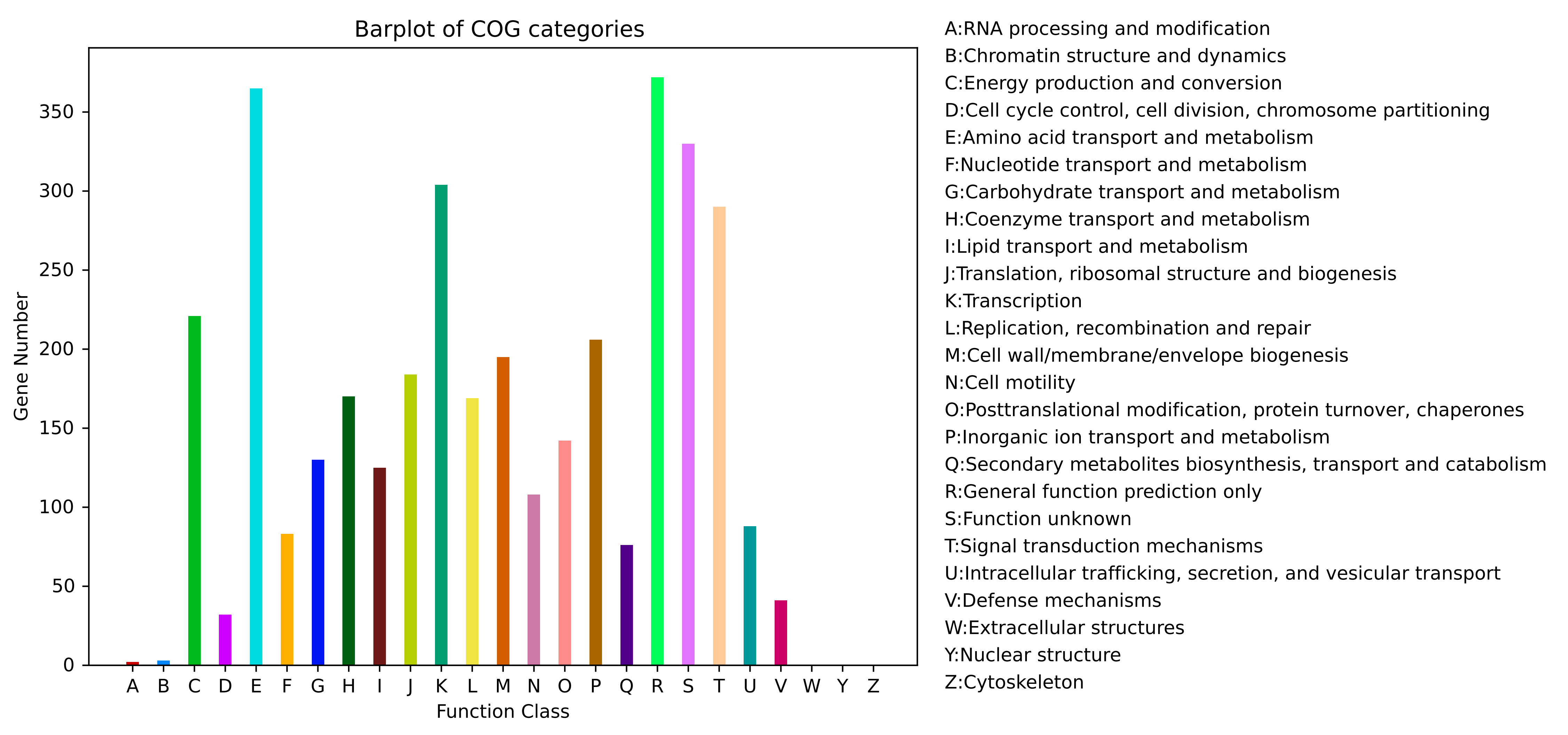

Barplot of COG categories
Gene Number
0
50
100
150
200
250
300
350
A B C D E F G H I J K L M N O P Q R S T U V W Y Z
Function Class
A:RNA processing and modification
B:Chromatin structure and dynamics
C:Energy production and conversion
D:Cell cycle control, cell division, chromosome partitioning
E:Amino acid transport and metabolism
F:Nucleotide transport and metabolism
G:Carbohydrate transport and metabolism
H:Coenzyme transport and metabolism
I:Lipid transport and metabolism
J:Translation, ribosomal structure and biogenesis
K:Transcription
L:Replication, recombination and repair
M:Cell wall/membrane/envelope biogenesis
N:Cell motility
O:Posttranslational modification, protein turnover, chaperones
P:Inorganic ion transport and metabolism
Q:Secondary metabolites biosynthesis, transport and catabolism
R:General function prediction only
S:Function unknown
T:Signal transduction mechanisms
U:Intracellular trafficking, secretion, and vesicular transport
V:Defense mechanisms
W:Extracellular structures
Y:Nuclear structure
Z:Cytoskeleton
