# Supplementary material for: Emergence of a multidrug-resistant Pseudomonas fulva clinical isolate co-harboring tmexCD3–toprJ3, blaOXA-1, and blaIMP-45 on a transferable megaplasmid
Source: Front Cell Infect Microbiol. 2026 Feb 16;16:1722020. doi: 10.3389/fcimb.2026.1722020 (PMC12950786; doi:10.3389/fcimb.2026.1722020)
Supplement: Supplementary file 8 [file Table2.docx]

Table S2. Comparison of antimicrobial susceptibility testing results before and after conjugation transfer experiments.

| Antimicrobial agent | PF1MIC^a^(μg/mL) | PA1MIC^a^(μg/mL) | TPA1MIC^a^(μg/mL) |
| --- | --- | --- | --- |
| Ticarcillin-Clavulanate | **>128（R）** | 64（I） | **>128（R）** |
| Piperacillin-Tazobactam | **>128（R）** | ≤4（S） | **>128（R）** |
| Cefoperazone-Sulbactam | **>64（R）** | ≤1（S） | **>64（R）** |
| ^b^Ceftazidime-Avibactam | **R** | S | **R** |
| Ticarcillin | **>128（R）** | **>128（R）** | **>128（R）** |
| Piperacillin | **>128（R）** | 16（S） | **>128（R）** |
| Aztreonam | 16（I） | ≤1（S） | 16（I） |
| Ceftazidime | **>64（R）** | 0.5（S） | **>64（R）** |
| Cefepime | **>32（R）** | ≤0.12（S） | **>32（R）** |
| Imipenem | **>16（R）** | 2（S） | **>16（R）** |
| Meropenem | **>16（R）** | ≤0.25（S） | **>16（R）** |
| Amikacin | **>64（R）** | ≤2（S） | **>64（R）** |
| Tobramycin | **>16（R）** | ≤1（S） | **>16（R）** |
| Levofloxacin | **>8（R）** | 1（S） | **>8（R）** |
| Ciprofloxacin | **>4（R）** | 1（I） | **>4（R）** |
| Norfloxacin | **>16（R）** | ≤0.5（S） | 8（I） |
| ^c^Tetracycline | 8（NA） | 1（NA） | 8（NA） |
| ^c^Doxycycline | 8（NA） | 4（NA） | 16（NA） |
| ^c^Minocycline | 8（NA） | 2（NA） | 16（NA） |
| ^c^Tigecycline | 256（NA） | 0.5（NA） | 64（NA） |
| ^c^Eravacycline | 4（NA） | 0.125（NA） | 2（NA） |
| ^c^Trimethoprim-Sulfamethoxazole | >320（NA） | ≤20（NA） | >320（NA） |
| Colistin | ≤0.5（S） | **16（R）** | **16（R）** |

^a^MICs above BrCAST/EUCAST clinical breakpoints are in bold.

^b^Ceftazidime-Avibactam were tested by the Kirby-Bauer disk diffusion method.

^c^Tetracycline, ^c^Doxycycline, ^c^Minocycline, ^c^Tigecycline, ^c^Eravacycline, and ^c^Trimethoprim-Sulfamethoxazole have no clinical breakpoints established for *Pseudomonas spp*. in either the CLSI or EUCAST guidelines; therefore, only MIC values were reported and no susceptibility categorization (S/I/R) was applied.

^d^PF1 was used as the donor strain, PA1 as the recipient strain, and TPA1 as the transconjugant.
